# Perceptions of orthodontic residents toward the implementation of dental technologies in postgraduate curriculum

**DOI:** 10.1186/s12903-023-03327-x

**Published:** 2023-09-01

**Authors:** Theerasak Nakornnoi, Chanchawan Chantakao, Nutchanon Luangaram, Thapakorn Janbamrung, Teetouch Thitasomakul, Kawin Sipiyaruk

**Affiliations:** 1 Department of Orthodontics, Faculty of Dentistry, Mahidol University, Bangkok, Thailand; 2 Doctor of Dental Surgery Program, Faculty of Dentistry, Mahidol University, Bangkok, Thailand

**Keywords:** Dental education, Digital dentistry, Orthodontics, Outcome-based education, Technology

## Abstract

**Background:**

Dental technologies have increasingly been implemented in orthodontic practice to offer better experiences for orthodontists and patients, however, there is no scientific evidence yet whether which technologies should be implemented into the postgraduate programs.

**Objectives:**

To investigate perceptions of orthodontic residents toward the confidence and importance of dental technologies, as well as to determine their necessity in postgraduate programs.

**Materials and methods:**

The online questionnaire was designed to collect data from residents from all accredited orthodontic postgraduate programs in Thailand. The questionnaire consisted of four sections, which were (1) demographic data, (2) self-perceived importance of orthodontic technologies, (3) self-perceived confidence toward orthodontic technologies, and (4) the necessity of orthodontic technologies in postgraduate programs. The data were analyzed using descriptive statistics, Spearman correlation, and a chi-square test.

**Results:**

Intraoral scanner was found to be an orthodontic technology with the highest scores for both self-perceived importance (4.37 ± 0.59) and confidence (4.23 ± 0.75), followed by cone-beam computed tomography, digital treatment planning software, and lab-produced aligners. These orthodontic technologies were also considered as mandatory in orthodontic postgraduate programs. CAD/CAM technologies appeared to be least important, and their training may be arranged as short course training. There was no significant influence of training locations on the necessity of all orthodontic technologies (*P* > 0.05), except CBCT. Self-perceived importance and confidence in all technologies were found to have significant positive correlations (*P* < 0.05), except teledentistry and in-office aligners.

**Conclusion:**

Orthodontic technologies were perceived as important in clinical workflow. Intraoral Scanners, CBCT, digital treatment planning software, lab-produced aligners, and digital modeling software appeared to be necessary for clinical practice and should be considered for orthodontic postgraduate programs, while other technologies may be arranged as short course training. Further research should investigate how to arrange and organize training sessions in orthodontic postgraduate programs.

**Supplementary Information:**

The online version contains supplementary material available at 10.1186/s12903-023-03327-x.

## Introduction

Dental technology has been increasingly employed in modern dentistry to improve the quality of treatment and working performance, by offering a wide range of benefits for both professionals and patients through faster processing, higher precision, and greater efficacy [1, 2]. Dental procedures currently are modernized through the implementation of technology such as digital radiography, impression taking, and treatment planning software [3–5]. These technologies have also been adopted in orthodontic practice to offer better experiences for orthodontists and patients, such as rapid result analysis and accurate treatment outcomes [6, 7]. Therefore, there seems to be an increasing use of technology-enhanced dental procedures in orthodontic practice.

Not only cone-beam computed tomography (CBCT) but also intraoral and facial scanning technology have become essential in orthodontic diagnosis and treatment planning, as these facilities can enhance a diagnostic workflow and patient communication through tooth movement simulations in order to create a virtual treatment plan [8–10]. They could offer 3D visualization to take the place of 2D traditional methods, where orthodontists could discuss virtual treatment outcomes with their patients. In addition, 3D printing technologies can be employed in clinical practice for orthodontic appliance fabrication [11, 12]. Clear aligners, robotically formed archwires, and custom brackets could be fabricated by computer-aided design/computer-aided manufacture (CAD/CAM) and 3D printing technology [13–17]. These orthodontic technologies can significantly reduce chair time of orthodontists with the enhancement of treatment quality.

Despite an increasing use of orthodontic technologies, there are challenges to adopt them in clinical practice. These barriers and difficulties may result from social and personal perceptions of orthodontic innovation [18]. In other words, technology adoption in orthodontic practice could be affected by the recognition of its importance among orthodontists and patients. In addition, the competence and confidence of orthodontists could have influence on how they would implement technology in their practice [19]. Consequently, to promote the use of orthodontic technologies, it is necessary to enhance both self-perceived importance and confidence among orthodontists.

Orthodontists can become more confident and competent in adopting technology through a number of training formats. Conferences, workshops, and continuing education courses can be arranged for orthodontists to learn about new technologies and how to use them effectively [20]. The implementation of technology training in orthodontic postgraduate programs could be another option, as it allows residents to feel more confident in their practice. However, no scientific evidence has yet reported whether which technologies should be implemented into postgraduate programs, where their timeframe for graduation is limited. Therefore, this research was conducted to investigate perceptions of orthodontic residents toward the importance and confidence of dental technologies, as well as to determine their necessity in postgraduate programs. The findings retrieved from this study would allow instructors or educators to design appropriate content of technology trainings for orthodontic curriculum.

## Materials and methods

This study employed a quantitative cross-sectional research design, using an online questionnaire as a data collection tool. The data collection process was conducted between October 2022 and February 2023. The ethical approval for the study was waived by the Institutional Review Board of Faculty of Dentistry and Faculty of Pharmacy, Mahidol University on 31st August 2022, the ethical approval number: MU-DT/PY-IRB 2022/040.3108.

The research participants were included if they were residents from all accredited orthodontic postgraduate programs in Thailand. However, they were excluded if they had trained in their programs for more than five years, as they might have had much experience with those technologies, compared to other respondents. The sample size was calculated using a formula for finite population. To achieve the 95% confidence interval with a margin of error at 4%, 108 orthodontic residents were expected to complete and return the questionnaire. Two reminders were sent at three-week intervals to enhance a response rate.

The questionnaire was constructed, based on previous literature regarding the use of technologies in orthodontic practice [21–23]. The questionnaire consisted of four sections, which were (1) demographic data, (2) self-perceived importance toward orthodontic technologies, (3) self-perceived confidence toward orthodontic technologies, and (4) the necessity of orthodontic technologies in postgraduate programs. Parts 2 and 3 were designed using a 5-point Likert scale, where 1 being not at all important/confident and 5 being very important/confident, while Part 1 and 4 were checklists (Supplementary material 1).

To assure the quality of questionnaire design, it was piloted to confirm its validity and reliability. Content validity was performed with three experts in orthodontic technologies, where the questionnaire was iteratively revised until each item achieved the index of item-objective congruence higher than 0.5. The validated version of the questionnaire was then piloted in 30 respondents to perform a test-retest reliability using an intraclass correlation coefficient (ICC), where the values of all items ranged from 0.73 to 0.91.

The data retrieved from the questionnaire were analyzed with the Statistical Package for Social Sciences software (SPSS, version 28, IBM Corp., Armonk, NY). Descriptive statistics were conducted to demonstrate an overview of the data. Spearman correlation was performed to determine correlations between self-perceived importance and confidence. The impact of training locations on the necessity of technologies in orthodontic curriculum was investigated with a chi-square test.

## Results

### Research respondents

There were 112 orthodontic residents who completed and returned the questionnaire, resulting in a response rate of 86.15%. Of these, 36 were males (32.14%), and 76 were females (67.86%). The average age of responders was 26.97 (SD = 0.28) years old. More than half of residents (53.57%) were training outside Bangkok (the capital city), while 46.43% were in Bangkok.

### Self-perceived importance and confidence toward orthodontic technologies

‘Intraoral scanner’ was found to be the highest score of both self-perceived importance (4.37 ± 0.59) and confidence (4.23 ± 0.75), followed by ‘CBCT’, ‘digital treatment planning software’, and ‘lab-produced aligners’. The respondents also tended to considered these four technologies to be trained as mandatory in orthodontic curricula, rather than short course training. ‘CAD/CAM brackets’ seemed to be the technology perceived by the respondents as least important and confident. These results were demonstrated in Table 1.

**Table 1:** Self-perceived importance, self-perceived confidence, and necessity of orthodontic technologies

Technology	Self-perceived importance Mean (SD)	Self-perceived confidence Mean (SD)	Training	
			Mandatory in curriculum n (%)	Short course training n (%)
**Intraoral scanners**	4.37 (0.59)	4.23 (0.75)	98 (87.5%)	14 (12.5%)
**CBCT**	4.29 (0.61)	3.77 (0.90)	100 (89.29%)	12 (10.71%)
**Digital treatment planning software**	4.27 (0.63)	3.64 (0.83)	102 (91.07%)	10 (8.93%)
**Lab-produced aligners**	4.20 (0.64)	3.64 (0.85)	100 (89.29%)	12 (10.71%)
**Digital modeling software**	4.03 (0.82)	3.29 (1.03)	91 (81.25%)	21 (18.75%)
**3D printing**	3.89 (0.84)	3.41 (0.95)	83 (74.11%)	29 (25.89%)
**In-office aligners**	3.88 (0.74)	3.16 (1.00)	94 (83.93%)	18 (16.07%)
**Teledentistry**	3.66 (0.82)	3.13 (0.87)	68 (60.71%)	44 (39.29%)
**Extraoral scanners**	3.49 (0.83)	3.24 (1.02)	73 (65.18%)	39 (34.82%)
**CAD/CAM wires**	3.36 (0.91)	2.75 (1.06)	66 (58.93%)	46 (41.07%)
**CAD/CAM brackets**	3.29 (0.94)	2.70 (1.05)	64 (57.14%)	48 (42.86%)

### Impact of training locations on necessity of dental technologies in orthodontic curriculum

Training locations were not found to have significant influence on the necessity of all orthodontic technologies in postgraduate curriculum (*P* > 0.05), except CBCT which was perceived by residents trained in the capital city as more necessary in their programs (*P* < 0.01), as presented in Table 2.

**Table 2:** Impact of training locations on necessity of orthodontic technologies

Technology	Training locations	Mandatory in curriculum (n)	Short course training (n)	*P*-value
**Intraoral scanners**	In the capital city	43	9	0.152
	Outside the capital city	55	5	
**Extraoral scanners**	In the capital city	29	23	0.052
	Outside the capital city	44	16	
**CBCT**	In the capital city	42	10	0.007
	Outside the capital city	58	2	
**3D printing**	In the capital city	40	12	0.527
	Outside the capital city	43	17	
**CAD/CAM wires**	In the capital city	31	21	0.891
	Outside the capital city	35	25	
**CAD/CAM brackets**	In the capital city	29	23	0.784
	Outside the capital city	35	25	
**Digital modeling software**	In the capital city	46	6	0.069
	Outside the capital city	45	15	
**Digital treatment planning software**	In the capital city	47	5	0.535
	Outside the capital city	55	5	
**Teledentistry**	In the capital city	30	22	0.542
	Outside the capital city	38	22	
**In-office aligner**	In the capital city	44	8	0.854
	Outside the capital city	50	10	
**Lab-produced aligners**	In the capital city	46	6	0.793
	Outside the capital city	54	6	

### Correlations between self-perceived importance and confidence

There were significant positive correlations between self-perceived importance and confidence in all technologies (*P* < 0.01), except teledentistry and in-office aligners. Correlation coefficients of all technologies demonstrated low to moderate correlations (Table 3).

**Table 3:** Correlations between self-perceived importance and confidence

Technology	Correlations between self-perceived importance and confidence	
	r	*P*-value
**Intraoral scanners**	0.362	0.000
**Extraoral scanners**	0.392	0.000
**CBCT**	0.339	0.000
**3D printing**	0.415	0.000
**CAD/CAM wires**	0.428	0.000
**CAD/CAM brackets**	0.481	0.000
**Digital modeling software**	0.412	0.000
**Digital treatment planning software**	0.279	0.003
**Teledentistry**	0.162	0.087
**In-office aligners**	0.154	0.102
**Lab-produced aligners**	0.314	0.001

## Discussion

Orthodontic technologies were considered as important by residents in clinical workflow, as demonstrated by self-perceived importance across all technologies, as they could offer rapid result analysis and accurate treatment outcomes [1, 18, 24]. Intraoral scanners were perceived by orthodontic residents as most important and confident. This technology has shown its efficiency in terms of higher accuracy and patient comfort compared to conventional approaches [6, 19, 25–27]. On the other hand, CAD/CAM brackets achieved the lowest rating in both perceptions. This technology was found to be recognized at a lower level compared to others due to their high cost and insignificant impact on clinical practice workflow [28–30]. In addition, there is evidence demonstrating a longer treatment duration (a number of visits) and questionable quality of treatment outcomes for orthodontic treatment with CAD/CAM bracket [31, 32]. Consequently, efficacy of clinical workflow and patient experiences appeared to be key considerations for orthodontists to adopt new orthodontic technologies for their practice.

Significant correlations between self-perceived importance and confidence were also revealed in most of the orthodontic technologies except teledentistry and in-office aligners. In other words, the respondents tended to rate their self-perceived confidence of an individual technology higher when they perceived it as more important. Teledentistry and in-office aligners have currently become important in orthodontic practice [33–36], however they might have not yet been implemented in postgraduate programs. There is evidence reporting that orthodontists tended to implement orthodontic technologies into their practices although they felt sensitive to potential obstacles if they recognized their benefits [37]. Experiences and perceived values can drive the motivation of adopters and then influence their decision to adopt a new technology [38]. Orthodontic residents with higher level of confidence and competence tended to implement digital technology in their practice [19]. Consequently, training of orthodontic technologies should be arranged in postgraduate curricula, allowing residents have more competence and confidence.

The majority of technologies were perceived as necessity in clinical practice, as residents tended to considered them as mandatory in orthodontic curricula. Although there is a possibility that participants who practiced in a capital city tended to have higher importance and confidence due to the disparity in technology accessibility between the city and remote areas [39, 40], training locations were not found to have impact on the necessity of all orthodontic technologies in this research, except CBCT. The residents who were trained outside the capital city perceived CBCT as more necessary than those trained in Bangkok. As there are very few dental offices with CBCT outside the capital city, the residents who were trained there might have been exposed to CBCT experiences. Consequently, CBCT was perceived as more necessary among orthodontic residents who were trained outside the capital city.

In terms of orthodontic education, the findings would allow instructors or educators to design appropriate technology trainings for their curriculum. With a limited timeframe of three-year period, orthodontic technologies could be selected from most to least important, according to the concept of outcome-based education [41]. For instance, intraoral scanners, CBCT, digital treatment planning software, lab-produced aligners, and digital modeling software appeared to be technologies required for orthodontic postgraduate programs. Digital treatment planning software or tooth movement simulation can also be implemented as an interactive learning tool [42]. These technological facilities can be considered as educational resource in the evaluation of curriculum quality [43]. Other technologies can be arranged as short course training, where orthodontists or residents may opt to attend based upon their interest.

This research purposed to gather information from orthodontic residents rather than orthodontists, as they were being trained in dental schools, so their responses could directly reflect the current practice of orthodontic curricula. However, orthodontic residents might not have global perspectives and awareness to consider the importance of these technologies, and therefore further research should extend a survey to experienced orthodontists. They would also be able to provide information regarding the limitations and necessity of digital orthodontics in clinical practice outside university settings. In addition, due to the limitation of quantitative research, qualitative studies should be further required to gather in-depth information to enhance the understanding of this topic. In addition, as this research revealed perceived importance and confidence of residents toward orthodontic technologies, further research should investigate appropriate training formats of these technologies.

## Conclusions

Orthodontic residents tended to perceive technologies as important in clinical workflow. However, each technology was not considered as important at the same level. Intraoral Scanners, CBCT, digital treatment planning software, lab-produced aligners, and digital modeling software appeared to be necessary, and their training should be arranged orthodontic postgraduate programs. Other orthodontic technologies may be arranged as short course training due to a limited timeframe of postgraduate curricula. However, further research should investigate how to effectively arrange and organize training sessions for residents to experience those orthodontic technologies in their clinical practice.

### Electronic supplementary material

Below is the link to the electronic supplementary material.

Supplementary Material 1

## Data Availability

The data that support the findings of this study are available from the corresponding author, upon reasonable request. The data are not publicly available due to information that could compromise the privacy of research participants.

## References

[1] van der Zande MM, Gorter RC, Bruers JJM, Aartman IHA, Wismeijer D (2018). Dentists’ opinions on using digital technologies in dental practice. Community Dent Oral Epidemiol.

[2] Christopoulou I, Kaklamanos EG, Makrygiannakis MA, Bitsanis I, Tsolakis AI (2021). Patient-reported experiences and preferences with intraoral scanners: a systematic review. Eur J Orthod.

[3] Francisco I, Ribeiro MP, Marques F, Travassos R, Nunes C, Pereira F (2022). Application of Three-Dimensional Digital Technology in Orthodontics: the state of the art. Biomimetics.

[4] Ronsivalle V, Ruiz F, Lo Giudice A, Carli E, Venezia P, Isola G (2023). From Reverse Engineering Software to CAD-CAM Systems: how Digital Environment has Influenced the clinical applications in Modern Dentistry and Orthodontics. Appl Sci.

[5] Sehrawat S, Kumar A, Grover S, Dogra N, Nindra J, Rathee S (2022). Study of 3D scanning technologies and scanners in orthodontics. Mater Today: Proc.

[6] Jacox LA, Mihas P, Cho C, Lin FC, Ko CC (2019). Understanding technology adoption by orthodontists: a qualitative study. Am J Orthod Dentofacial Orthop.

[7] Impellizzeri A, Horodynski M, De Stefano A, Palaia G, Polimeni A, Romeo U (2020). CBCT and Intra-Oral scanner: the advantages of 3D Technologies in Orthodontic Treatment. Int J Environ Res Public Health.

[8] Park JH, Lee G-H, Moon D-N, Yun K-D, Kim J-C, Lee KC (2022). Creation of Digital virtual patient by integrating CBCT, Intraoral scan, 3D facial scan: an Approach to Methodology for Integration Accuracy. J Craniofac Surg.

[9] Urban R, Haluzová S, Strunga M, Surovková J, Lifková M, Tomášik J (2023). AI-Assisted CBCT Data Management in Modern Dental Practice: benefits, Limitations and Innovations. Electronics.

[10] Cattaneo PM, Cornelis MA (2023). Digital Workflows in Orthodontic Postgraduate Training. Semin Orthod.

[11] Graf S, Tarraf NE, Vasudavan S (2022). Direct printed removable appliances: a new approach for the twin-block appliance. Am J Orthod Dentofacial Orthop.

[12] Federici Canova F, Oliva G, Beretta M, Dalessandri D (2021). Digital (R)evolution: open-source softwares for Orthodontics. J Appl Sci.

[13] Brown MW, Koroluk L, Ko CC, Zhang K, Chen M, Nguyen T (2015). Effectiveness and efficiency of a CAD/CAM orthodontic bracket system. Am J Orthod Dentofacial Orthop.

[14] Nguyen T, Jackson T (2018). 3D technologies for precision in orthodontics. Semin Orthod.

[15] Khosravi R, Gidarakou I, Salazar T (2022). Essential factors in developing an efficient in-office aligner system. Semin Orthod.

[16] Tsolakis IA, Gizani S, Tsolakis AI, Panayi N (2022). Three-dimensional-printed customized Orthodontic and Pedodontic Appliances: a critical review of a new era for treatment. Children.

[17] Goracci C, Juloski J, D’Amico C, Balestra D, Volpe A, Juloski J (2023). Clinically relevant Properties of 3D printable materials for Intraoral Use in Orthodontics: a critical review of the literature. Materials.

[18] van der Zande MM, Gorter RC, Wismeijer D (2013). Dental practitioners and a digital future: an initial exploration of barriers and incentives to adopting digital technologies. Br Dent J.

[19] Rehil S, Subramani K, Sinha P, Jain S, Roberson G, Chattopadhyay A (2022). Education using clear aligners and digital workflows in graduate orthodontic residency programs. Semin Orthod.

[20] Jacox LA, Bocklage C, Edwards T, Mihas P, Lin F-C, Ko C-C (2022). Understanding technology adoption by orthodontists: a quantitative study. Am J Orthod Dentofacial Orthop.

[21] Leonardi RM (2022). 3D imaging advancements and New Technologies in Clinical and Scientific Dental and Orthodontic Fields. J Clin Med.

[22] Vaid NR (2018). Digital technologies in orthodontics–An update. Semin Orthod.

[23] Tarraf NE, Ali DM (2018). Present and the future of digital orthodontics. Semin Orthod.

[24] Elnagar MH, Aronovich S, Kusnoto B (2020). Digital Workflow for combined orthodontics and orthognathic surgery. Oral Maxillofac Surg Clin North Am.

[25] Kihara H, Hatakeyama W, Komine F, Takafuji K, Takahashi T, Yokota J (2020). Accuracy and practicality of intraoral scanner in dentistry: a literature review. J Prosthodont Res.

[26] Burzynski JA, Firestone AR, Beck FM, Fields HW, Deguchi T (2018). Comparison of digital intraoral scanners and alginate impressions: Time and patient satisfaction. Am J Orthod Dentofacial Orthop.

[27] Aizenbud D, Hazan-Molina H, Zere E, Aizenbud N, Aizenbud Y (2021). Intraoral iTero scanning for an infant with cleft lip and palate. Am J Orthod Dentofacial Orthop.

[28] Tran D, Nesbit M, Petridis H (2016). Survey of UK dentists regarding the use of CAD/CAM technology. Br Dent J.

[29] Ardila CM, Elorza-Durán A, Arrubla-Escobar D (2022). Efficacy of CAD/CAM technology in interventions implemented in Orthodontics: a scoping review of clinical trials. Biomed Res Int.

[30] Czolgosz I, Cattaneo PM, Cornelis MA (2020). Computer-aided indirect bonding versus traditional direct bonding of orthodontic brackets: bonding time, immediate bonding failures, and cost-minimization. A randomized controlled trial. Eur J Orthod.

[31] Papakostopoulou M, Hurst D (2018). Customised fixed appliance systems and treatment duration. Evid Based Dent.

[32] Penning EW, Peerlings RHJ, Govers JDM, Rischen RJ, Zinad K, Bronkhorst EM (2017). Orthodontics with customized versus noncustomized appliances: a Randomized Controlled Clinical Trial. J Dent Res.

[33] Park JH, Kim JH, Rogowski L, Al Shami S, Howell SEI (2021). Implementation of teledentistry for orthodontic practices. J World Fed Orthod.

[34] Bentson C, Copple D (2022). Opportunities in the evolving orthodontic industry–digital processes, teledentistry and group practices. Semin Orthod.

[35] Roberson GA, Sinha PK (2022). 3D printing in orthodontics: a practical guide to the printer technology and selection. Semin Orthod.

[36] Maspero C, Abate A, Cavagnetto D, El Morsi M, Fama A, Farronato M (2020). Available Technologies, Applications and benefits of teleorthodontics. A literature review and possible applications during the COVID-19 pandemic. J Clin Med.

[37] Palmer NG, Yacyshyn JR, Northcott HC, Nebbe B, Major PW (2005). Perceptions and attitudes of canadian orthodontists regarding digital and electronic technology. Am J Orthod Dentofacial Orthop.

[38] Patel RN, Antonarakis GS (2013). Factors influencing the adoption and implementation of teledentistry in the UK, with a focus on orthodontics. Community Dent Oral Epidemiol.

[39] Jo O, Kruger E, Tennant M (2020). Geospatial analysis of the urban and rural/remote distribution of dental services in Scotland, Wales and Northern Ireland. Int Dent J.

[40] Soegyanto AI, Wimardhani YS, Maharani DA, Tennant M (2022). Indonesian dentists’ perception of the Use of Teledentistry. Int Dent J.

[41] Clark JD, Robertson LJ, Harden RM (2004). The specification of learning outcomes in dentistry. Br Dent J.

[42] Sipiyaruk K, Kaewsirirat P, Santiwong P (2023). Technology-enhanced simulation-based learning in orthodontic education: a scoping review. Dent Press J Orthod.

[43] Burad M, Laowanichwith C, Kiatsukasem A, Supa-amornkul S, Sipiyaruk K (2022). Conceptual Framework for implementation of internationalization in Dental Education with Foundations in Dental Student Life. Int J Environ Res Public Health.

