# Supplementary material for: Perceptions of orthodontic residents toward the implementation of dental technologies in postgraduate curriculum
Source: BMC Oral Health. 2023 Sep 1;23:625. doi: 10.1186/s12903-023-03327-x (PMC10474673; doi:10.1186/s12903-023-03327-x)
Supplement: Supplementary file 1 — Supplementary Material 1 [file 12903_2023_3327_MOESM1_ESM.docx]

**Questionnaire survey on perceptions toward the implementation of dental technologies in orthodontic curriculum**

**__________________________________________________________**

**Part 1: Demographics**

1. What is your sex?

- Male
- Female

2. What is your age? _________

3. How do you describe the location of your training?

- In the capital city
- Outside the capital city

**Part 2: Self-perceived importance toward orthodontic technologies**

Please rate each of the following items based on your perceptions toward the importance of dental technologies in orthodontic practice.

| Technology | 1  Not at all important | 2  Slightly important | 3  Important | 4  Fairly  important | 5  Very important |
| --- | --- | --- | --- | --- | --- |
| Intraoral scanners |  |  |  |  |  |
| Extraoral scanners |  |  |  |  |  |
| CBCT |  |  |  |  |  |
| 3D printing |  |  |  |  |  |
| CAD/CAM wires |  |  |  |  |  |
| CAD/CAM brackets |  |  |  |  |  |
| Digital modeling software |  |  |  |  |  |
| Digital treatment planning software |  |  |  |  |  |
| Teledentistry |  |  |  |  |  |
| In-office aligners |  |  |  |  |  |
| Lab-produced aligners |  |  |  |  |  |

**Part 3: Self-perceived confidence toward orthodontic technologies**

Please rate each of the following items based on your perceptions toward your confidence in the use of dental technologies in orthodontic practice.

| Technology | 1  Not at all confident | 2  Slightly confident | 3  Confident | 4  Fairly  confident | 5  Very confident |
| --- | --- | --- | --- | --- | --- |
| Intraoral scanners |  |  |  |  |  |
| Extraoral scanners |  |  |  |  |  |
| CBCT |  |  |  |  |  |
| 3D printing |  |  |  |  |  |
| CAD/CAM wires |  |  |  |  |  |
| CAD/CAM brackets |  |  |  |  |  |
| Digital modeling software |  |  |  |  |  |
| Digital treatment planning software |  |  |  |  |  |
| Teledentistry |  |  |  |  |  |
| In-office aligners |  |  |  |  |  |
| Lab-produced aligners |  |  |  |  |  |

**Part 4: The necessity of orthodontic technologies in postgraduate training**

Please rate each of the following items based on your perceptions toward the necessity of dental technologies in orthodontic postgraduate training.

| Technology | Mandatory in curriculum | Short course training |
| --- | --- | --- |
| Intraoral scanners |  |  |
| Extraoral scanners |  |  |
| CBCT |  |  |
| 3D printing |  |  |
| CAD/CAM wires |  |  |
| CAD/CAM brackets |  |  |
| Digital modeling software |  |  |
| Digital treatment planning software |  |  |
| Teledentistry |  |  |
| In-office aligners |  |  |
| Lab-produced aligners |  |  |
